# The relationship between levels of self-esteem and the development of depression in young adults with mild depressive symptoms

**DOI:** 10.1097/MD.0000000000017518

**Published:** 2019-10-18

**Authors:** Yoobin Choi, Soo-Hee Choi, Je-Yeon Yun, Jae-A Lim, Yoonhee Kwon, Hwa Young Lee, Joon Hwan Jang

**Affiliations:** a Department of Psychiatry, Seoul National University Hospital, Seoul,; b Seoul National University Hospital,; c Yeongeon Student Support Center, Seoul National University College of Medicine,; d Department of Psychiatry, Seoul National University Health Service Center,; e Department of Medicine, Seoul National University College of Medicine, Seoul, Republic of Korea.

**Keywords:** mild depressive symptom, resilience, self-esteem, social support, young adult

## Abstract

Little is known about the relationship between levels of self-esteem and the development of depression in young adults. The present study investigated the relationship between self-esteem and depression to determine whether self-esteem levels are a risk factor for the development of depression in young adults. This study was conducted with 113 college students aged 19 to 35 (major depressive disorder (MDD) n = 44, Mild Depressive Symptoms (MDS) n = 37, Healthy Control n = 32). The levels of clinical symptoms, self-esteem, resilience, social support, and quality of life, as well as personality traits, were assessed (by Patient Health Questionnaire-9, generalized anxiety disease-7, State-Trait Anxiety Inventory-S, Resilience Appraisal Scale, Rosenberg Self-Esteem Scale, Quality of Life, and NEO-personality inventory (NEO-PI)). The MDS group with high self-esteem reported having the lowest levels of social support, resilience, agreeableness, and extraversion compared to those of the MDD group and control group with high self-esteem. In contrast, the MDS group with low self-esteem showed no differences in social support, resilience, agreeableness and openness according to the NEO-PI scale. Sex and age had no significant impact on the results. Levels of self-esteem are strongly associated with the development of depression. Results suggest that early intervention for depression in young adults needs to focus on improving their levels of social support, resilience, and positive domains of personality. Further studies on the effects of high self-esteem in the development of depression are warranted.

## 1 Introduction

In the college student population, depression is a common disorder that has significant impacts on cognitive thinking, academic performance, relationships with peers, death rates, and self-esteem.^[1–3]^ About 20% to 30% of college students not receiving psychiatric services have experienced significant depressive symptoms.^[4]^ Although depression is associated with many problems, young adults often do not seek treatment and do not function well in their daily lives.^[5]^ Symptoms range widely from mild feelings of depression to very severe and deep feelings of depression. Many young adults with depressive symptoms are unaware of their symptoms at the beginning but may go on to experience suicidal thoughts.^[6]^ Therefore, studying the characteristics of mild depressive symptoms (MDS) in young adults is important for appropriate early intervention and improved prognosis.

Studies have revealed that self-esteem plays an important role in depression.^[7]^ Self-esteem is defined as “a certain attitude and a perception of one's self”,^[8]^ which affects interactions and feelings towards oneself and others. Self-esteem is also related to social support.^[9]^ Considering the relationship between depressive symptoms and interpersonal problems in young adults, it is important to study how self-esteem is affected by the development of depression. Orth et al,^[3]^ in a study of early adolescents, reported that self-esteem is a prospective risk factor for depression. Similarly, according to a previous study,^[10]^ respondents who had a spouse with low self-esteem had a higher depression score. In contrast, respondents who had a spouse with higher self-esteem had a lower depression score, regardless of sex. This result suggests that low self-esteem is related to depressive symptoms and social support is one of the most important factors affecting depressive symptoms. However, a recent study reported that high self-esteem is not always healthy.^[11]^ Kernis at the University of Georgia studied 100 undergraduates to investigate significant difference between students with “fragile” high self-esteem and students with “secure” high self-esteem. After determining their self-esteem levels through questionnaires and interviews, researchers found that people with secure high self-esteem were able to accept their negative traits more easily and were less likely to be verbally defensive. In contrast, students with fragile high self-esteem were verbally defensive, reflecting mental problems such as depression and anxiety. This study revealed that there are different types of high self-esteem and sometimes high self-esteem may lead to depressive symptoms. The relationship between self-esteem and the development of depression has not been studied yet; in particular, there has been no report on the effect of levels of self-esteem on MDS.

The present study investigated the relationship between self-esteem and depression, to determine whether the level of self-esteem is a risk factor for the development of depression in young adults. Previous studies have mainly focused on major depressive disorder versus healthy control subjects and have focused on either adults or younger age groups. The present study identified the clinical variables, according to the level of self-esteem, for young adults with MDS before symptoms became severe, to inform prevention and coordinate early treatment programs. We investigated clinical variables in college students with major depressive MDD, MDS, or healthy controls (CON), to clarify the effect of self-esteem levels on the development of depression. The groups were compared (according to the level of self-esteem) to determine cognitive or environmental factors affecting the MDS group. It was hypothesized that self-esteem levels may affect interpersonal interaction, social support, and stress resilience in the MDS state and may be a marker for depression.

## 2 Methods

### 2.1 Participants

A total of 114 (69 females, 45 males) undergraduate and graduate students, aged from 19 to 35 participated (mean age: male 25.08, female 24.11). Of the 114 participants, 1 was excluded due to use of a psychotropic medication before enrollment, resulting in a sample of 113. Exclusionary criteria included a history of psychosis, substance abuse or dependence, intellectual development disorder, or use of psychotropic medication within the previous 8 weeks before enrollment.

The participants with clinical depressive symptoms were recruited via electronic mail and from the mental health clinic in Seoul National University after they sought treatment. They met at least one of the following criteria:

Patient Health Questionnaire-9 ≥ 10 points

generalized anxiety disease-7 ≥ 10 points

State-Trait Anxiety Inventory-State ≥ 61 points (for males) or ≥ 65 points (for females)

at least 1 suicidal thought/attempt/plan within the past 6 months.

Additionally, the subjects had either

depressed mood or

loss of interest or pleasure.

Then, the participants with clinical depressive symptoms were divided into either the MDD or MDS group. The MDD group consisted of 32 participants meeting the DSM-5 criteria for MDD. The remaining 37 participants were classified as the MDS group. CON groups were recruited by flyers. Absence of Axis I psychiatric disorders in healthy controls were confirmed with the Mini-International Neuropsychiatric Interview (MINI).

The present study was approved by the Institutional Review Board at Seoul National University College of Medicine and Hospital (Seoul, Republic of Korea; No. 1608-079-785). All participants received information about the purpose and content of the study, the expected time required, how privacy would be protected, and were allowed to quit the study any time.

### 2.2 Instruments

#### 2.2.1 Patient Health questionnaire-9 (PHQ-9)

The PHQ-9 was used to assess depression using a 9-item self-reported questionnaire with a 4-point Likert scale.^[12,13]^ The questionnaire included, for example, a question about the number of times participants had suffered from fear, memory problems, insomnia, and difficulties with interpersonal relationships over the last 2 weeks. The final score ranged from 0 to 27, with higher scores indicating higher depressive symptoms.

#### 2.2.2 Generalized anxiety disease-7 (GAD-7)

The GAD-7 was used to assess severity of generalized anxiety using a 7-item self-reported questionnaire with a 4-point scale.^[14]^ The GAD-7 included, for example, a question about the number of times the participants had suffered from uneasy feelings over the last two weeks. The final scores ranged from 0 to 21, with the higher scores indicating higher anxiety symptoms.

#### 2.2.3 State-trait anxiety inventory-state anxiety (STAI-S)

The STAI-S was used to measure the degree of anxiety using a 20-item self-reported questionnaire with a 4-point scale.^[15]^ The inventory included, for example, a question about whether the participants felt difficulty in daily life due to excessive anxiety. The final scores ranged from 20 to 80, with higher scores indicating higher anxiety symptoms.

#### 2.2.4 Rosenberg self-esteem scale (RSES)

The Korean version of RSES was used to assess self-esteem using a 10-item self-reported questionnaire with a four-point scale.^[16]^ The questions focus on how participants feel about the self by measuring both positively formulated and negatively formulated items, with high scores indicating higher self-esteem.

#### 2.2.5 Resilience appraisal scale (RAS)

The Korean version of RAS was used to assess resilience and to measure the ability to cope with emotions, solve problems, and gain social support. The RAS contains 3 types of positive self-appraisals, including emotion coping appraisals, situation coping appraisals, and social support appraisals.^[17]^ It has a 12-item self-reported questionnaire with a 5-point scale. The questions are about participants’ emotional control and their relationships with friends and family. The final score ranged from 12 to 60, with higher scores indicating higher resilience.

#### 2.2.6 Social support scale

The social support scale was used to measure perceptions of social support and satisfaction with interpersonal relationship using 25-item self-reported questionnaire.^[18]^ Each item is rated by a five-point scale, consisting of emotional, informational, and material support, and evaluation of such support. The final scores ranged from 25 to 125, with higher scores indicating higher levels of social support.

#### 2.2.7 World health organization quality of life-bref (WHOQOL-BREF)

The Korean version of WHOQOL-BREF includes domains of Physical Health, Psychological State, Social Relationships, and the Environment.^[19,20]^ It is rated by a 5-point scale, consisting of a 26-item self-reported questionnaire. The questionnaire included questions about satisfaction and happiness based on experiences over the past 2 weeks. The final score ranged from 26 to 130, with higher scores indicating higher quality of life.

#### 2.2.8 NEO personality inventory (NEO-PI)

The NEO-PI provides a general description of personality traits and was used to assess personality traits using a 60-item self-reported questionnaire with a 5-point scale.^[21]^ Based on the 5- factor model, the NEO-PI measures the interpersonal, motivational, and emotional styles of both adults and adolescents. The 5 factors include: Openness to Experience, Conscientiousness, Extraversion, Agreeableness, and Neuroticism.

### 2.3 Statistical analyses

First, we used Descriptive Statistics to analyze the demographic and clinical characteristics of participants. Then, the Chi-Square test was performed to assess the distribution of the 3 groups according to the self-esteem classification based on the cutoff point. Lastly, we conducted a 1-way analysis of variance (ANOVA) with the Bonferroni post hoc test to evaluate which variables at the level of self-esteem could be used to identify the MDS group among the 3 severity groups (MDD, MDS, CON). A *P* value of *P* < .05 was considered to be statistically significant. All analyses were conducted using version 23 of IBM SPSS.

## 3 Results

### 3.1 Demographic and clinical characteristics

Table 1 shows the means and standard deviations of clinical and demographic variables. There was no significant difference between the 3 groups in terms of sex and age. However, there were significant differences between the three groups in all clinical variable scores (*P* < .01 for all variables), except clinical variables of the NEO-PI openness score (*P* = .528). Based on the scores, the MDS group is significantly more relevant to the MDD group than the CON group.

**Table 1:**
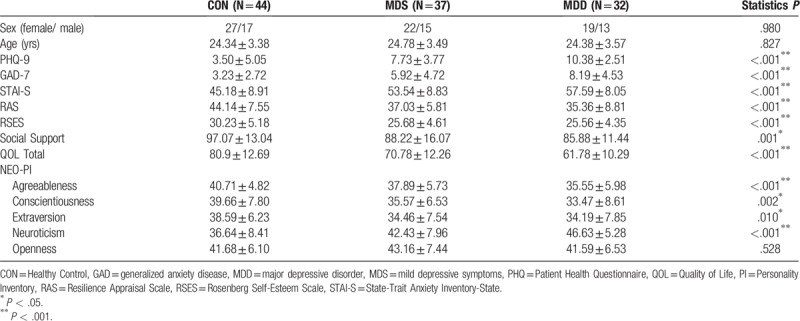
The demographic and clinical characteristics of participants.

### 3.2 Self-esteem classification based on the cutoff points

Table 2 shows the distribution of the groups according to the self-esteem classification. We categorized the total score of RSES as low (10–28) or high (29 to 40) level, with the cut-off point set at 29.^[22]^ Based on the cut-off point, the percentage with high self-esteem was 61.4% in the CON group, 29.7% in the MDS group, and 31.3% in the MDD group. The percentage with low self-esteem was 38.6% in the CON group, 70.3% in the MDS group, and 68.8% in the MDD group. Overall, these results indicate that the CON group had the highest self-esteem and the MDS group had the lowest self-esteem. With these results, we identified specific variables that mediate the MDS group according to the level of self-esteem.

**Table 2:**
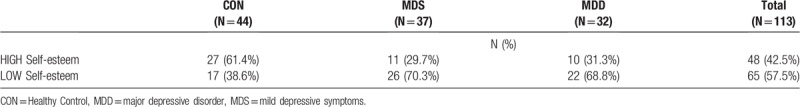
Distribution of groups according to the self-esteem classification based on the cut-off points.

### 3.3 Clinical variables according to the level of self-esteem

As demonstrated in Table 3, an analysis of variables revealed significant differences in clinical variables, except for the NEO-PI openness score, in both the high self-esteem group and low self-esteem group.

**Table 3:**
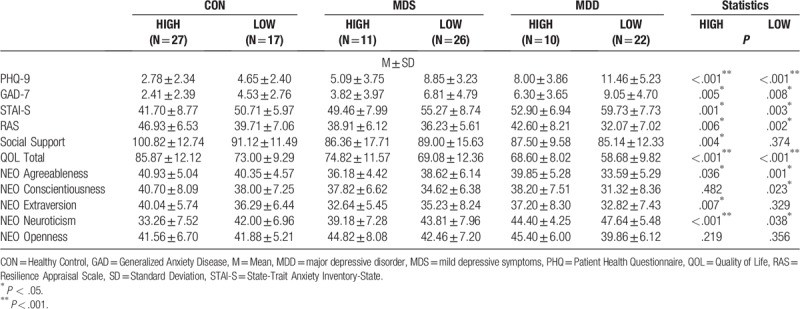
The mean scores and standard deviations of all clinical variables according to the level of self-esteem.

When comparing the difference between high and low self-esteem in the three groups using a post-hoc analysis, a one-way ANOVA demonstrated that the difference in clinical variables at the level of self-esteem was statistically highly significant among the three groups. Furthermore, post-hoc comparison using Bonferroni's correction indicated that the MDS group with high self-esteem had the lowest score of resilience (38.91 ± 6.12; *P* = .006) and social support (86.36 ± 17.71; *P* = .004), compared to the MDD group and control group with high self-esteem. Additionally, the mean score of the MDS group with high self-esteem was significantly lower than the MDD group and the CON group with high self-esteem. In addition, agreeableness (36.18 ± 4.42; *P* = .036) and extraversion (32.64 ± 5.45; *P* = .007) in the NEO personality trait scale were low compared to the MDD group and the CON group with high self-esteem. The mean score of the MDS group with high self-esteem was significantly lower than the MDD group and the CON group with high self-esteem.

On the other hand, after using Bonferroni's correlations between all clinical variables of the 3 groups with low self-esteem, the MDS group with low self-esteem showed no difference in resilience, social support, and agreeableness and openness on the NEO-PI scale. Even though the MDS group with low self-esteem reported a higher level of openness than those of the MDD group and CON group with low self-esteem, there was no significant difference between the 3 groups (*P* = .356). The overall mean score of the MDS group did not significantly discriminate the characteristics of the MDS group.

## 4 Discussion

The aim of the present study was to examine the relationship between the level of self-esteem and depression, to evaluate which clinical variables, at the different levels of self-esteem, could predict the development of depression among the 3 severity groups. In line with our first hypothesis, we found that the MDS group was significantly more relevant to the MDD group than the CON group. Although many antidepressant medications are useful and appear to be effective for MDD, there are several limitations and side effects with antidepressant medications used to treat mild depression.^[5]^ These results suggest that it is important to find the factors that mediate mild depression symptoms.

Moreover, we identified several predictors of mild depression in subjects aged 19 to 35. First, the MDS group with high self-esteem reported having the lowest level of social support among the 3 groups, indicating poor-quality relationships and lack of emotional support. Previous research supports this result that social isolation and lack of social support are important risk factors for depression.^[23]^

The MDS group with high self-esteem also reported having the lowest level of resilience. In line with results from an earlier report that self-esteem had a positive prospective effect on social support,^[3]^ this result suggests that a lack of social support may decrease resilience and lead to depression in young adults with high self-esteem. These findings are consistent with past research that reported a strong association between low resilience and probable depression, where psychological resilience was strongly linked to social support.^[24]^ In the field of personality, the MDS group with high self-esteem had the lowest score on agreeableness and extraversion compared to the MDD and CON group with high self-esteem. People with low levels of extraversion are less outgoing, lack social support, and lack social interaction with others. People with low levels of agreeableness are suspicious, irritable, uncooperative, and unlikely to reach out to people.^[25]^ Previous research reported that high levels of neuroticism and low levels of agreeableness and extraversion were associated with depression and are risk factors for major depression.^[26]^ This result suggests that participants with low levels of agreeableness and extraversion could be prone to developing depression or other psychological distress. However, our result differs from other research that found negative self-evaluations are related to depression.^[27]^ Another previous study reported that low self-esteem can be a risk factor for developing depression and positive self-evaluation alleviates depressive symptoms.^[7]^ However, this study was conducted to identify the effectiveness of memory training only for individuals with low self-esteem in a state of depression. Overall, our findings reveal that a lack of social support may decrease resilience and lead to depression in young adults with high self-esteem.

There are several limitations to the present study. First, the sample size is relatively small. However, we found significant relationships from the data, which support our results. Second, the analyses were based on self-reported instruments. Even though self-reported questionnaires may not be entirely valid, half of the questions were reversed to avoid response bias and to protect anonymity and confidentiality. Last, the measures we used to collect the data are not sufficient to conduct a thorough analysis of the results. To enable a strong conclusion, a self-efficacy questionnaire should have been included. A previous study found that those with low self-efficacy and high perfectionism had increased depression levels.^[28]^ However, we have provided reliable evidence regarding risk factors and have examined the significant relationship between self-esteem and depression. The present study strengthens the conclusion that social support is very important for young adults with mild depressive symptoms.

To our knowledge, the present study is the first to examine the relationship between self-esteem and minor depressive symptoms in young adults, classified into three different groups (MDD, MDS, and CON group). The results reveal the relationship between levels of self-esteem and development of depression, suggesting that further studies are needed on the effects of high self-esteem on mild depression. In addition, early intervention for depression in young adults should be focused on identifying the characteristics of mild depressive symptoms and improving their levels of social support and resilience, to mediate the effect of self-esteem on the development of depression.

## Author contributions

**Conceptualization:** Yoobin Choi, Soo-Hee Choi, Joon Hwan Jang.

**Formal analysis:** Yoobin Choi, Soo-Hee Choi, Joon Hwan Jang.

**Investigation:** Yoobin Choi, Je-Yeon Yun, Jae-A Lim, Yoonhee Kwon, Hwa Young Lee.

**Methodology:** Yoonhee Kwon.

**Supervision:** Joon Hwan Jang.

**Writing – original draft:** Yoobin Choi, Soo-Hee Choi.

**Writing – review & editing:** Je-Yeon Yun, Joon Hwan Jang.

## References

[1] Hysenbegasi A Hass SL Rowland CR The impact of depression on the academic productivity of university students. J Ment Health Policy Econ 2005;8:145 51. 16278502

[2] Farabaugh A Bitran S Nyer M Depression and suicidal ideation in college students. Psychopathology 2012;45:228 34. 22627683 10.1159/000331598

[3] Orth U Robins RW Widaman KF Is low self-esteem a risk factor for depression? Findings from a longitudinal study of Mexican-origin youth. Dev Psychol 2014;50:622 33. 23895172 10.1037/a0033817 PMC3815504

[4] Yun JY Choi Y Kwon Y Hubness of strategic planning and sociality influences depressive mood and anxiety in College Population. Sci Rep 2017;7:17856. 29259322 10.1038/s41598-017-18189-x PMC5736715

[5] Kasper S Caraci F Forti B Efficacy and tolerability of Hypericum extract for the treatment of mild to moderate depression. Eur Neuropsychopharmacol 2010;20:747 65. 20708905 10.1016/j.euroneuro.2010.07.005

[6] Ross SM Mild to moderate depression: a complementary and integrative therapies approach. Holist Nurs Pract 2010;24:303 9. 20651600 10.1097/HNP.0b013e3181f103e4

[7] Korrelboom K Maarsingh M Huijbrechts I Competitive memory training (COMET) for treating low self-esteem in patients with depressive disorders: a randomized clinical trial. Depress Anxiety 2012;29:102 10. 22495940 10.1002/da.20921

[8] Mruk CJ Self-Esteem Research, Theory, and Practice: Toward a Positive Psychology of Self-Esteem. 3rd ed New York, NY: Springer Publishing Company; 2006.

[9] DuBois DL Burk-Braxton C Swenson LP Getting by with a little help from self and others: Self-esteem and social support as resources during early adolescence. Dev Psychol 2002;38:822 39. 12220058 10.1037//0012-1649.38.5.822

[10] Lee TH Kim TH Kim W Effects of difference in self-esteem between spouses on depressive symptom: Result from a data nationally representative of South Korean. Psychiatry Res 2016;246:623 8. 27839825 10.1016/j.psychres.2016.10.079

[11] Sciencedaily. High Self-esteem is not always what it's cracked up to be. 2008 Available at: www.sciencedaily.com/releases/2008/04/080428084235.htm Accessed November 1, 2018.

[12] Kroenke K Spitzer RL Williams JB The PHQ-9: validity of a brief depression severity measure. J Gen Intern Med 2001;16:606 13. 11556941 10.1046/j.1525-1497.2001.016009606.x PMC1495268

[13] Park SJ Choi HR Choi JH Reliability and validity of the Korean version of the Patient Health Questionnaire-9 (PHQ-9). Anxiety Mood 2010;6:119 24.

[14] Spitzer RL Kroenke K Williams JB A brief measure for assessing generalized anxiety disorder: the GAD-7. Arch Intern Med 2006;166:1092 7. 16717171 10.1001/archinte.166.10.1092

[15] Spielberg CD Gorsuch RL Lushene RE Manual for the State-Trait Anxiety Inventory. Palo Alto, CA: Consulting Psychologists Press; 1970.

[16] Rosenberg M Conceiving the Self. New York, NY: Basic Books; 1979.

[17] Johnson J Gooding PA Wood AM Resilience as positive coping appraisals: testing the schematic appraisals model of suicide (SAMS). Behav Res Ther 2010;48:179 86. 19906364 10.1016/j.brat.2009.10.007

[18] Yu EK Seol HS Factorial structure of the social support scale. Survey Res 2015;16:155 84.

[19] World Health Organization. WHOQOL-BREF: Introduction, Administration, Scoring and Generic Version of the Assessment. Geneva: World Health Organization; 1996.

[20] Min SK Kim KI Lee CI Development of Korean version of WHO quality of life scale abbreviated version (WHOQOL-BREF). J Korean Neuropsychiatr Assoc 2000;39:571 9.

[21] Ahn CK Chae JH Standardization of the Korean version of the revised NEO personality inventory. Korean J Couns Psychother 1997;9:443 72.

[22] Terra FS Marziale MH Robazzi ML Evaluation of self-esteem in nursing teachers at public and private universities. Rev Lat Am Enfermagem 2013;21:71 8. 23459893 10.1590/s0104-11692013000700010

[23] Allchin A Melchior M Fombonne E Parental social networks during childhood and offspring depression in early adulthood: a lifecourse approach. Depress Anxiety 2016;33:1031 8. 27373544 10.1002/da.22538

[24] Toukhsati SR Jovanovic A Dehghani S Low psychological resilience is associated with depression in patients with cardiovascular disease. Eur J Cardiovasc Nurs 2017;16:64 9. 26984970 10.1177/1474515116640412

[25] Rey L Extremera N Agreeableness and interpersonal forgiveness in young adults: the moderating role of gender. Terapia psicológica 2016;34:103 10.

[26] Afshar H Roohafza H Hassanzadeh-Keshteli A Association of personality traits with psychological factors of depression, anxiety, and psychological distress: a community based study. Int J Body Mind Culture 2015;2:105 14.

[27] Harter S Marold DB Whitesell NR Model of psychosocial risk factors leading to suicidal ideation in young adolescents. Dev Psychopathol 1992;4:167 88.

[28] Lee MK Kim GH The effect of perfectionism and self-efficacy on depression and task performance. Kor J Clin Psychol 1998;17:211 22.

